# Sustainable Cotton Gin Waste/Polycaprolactone Bio-Plastic with Adjustable Biodegradation Rate: Scale-Up Production through Compression Moulding

**DOI:** 10.3390/polym15091992

**Published:** 2023-04-23

**Authors:** Zengxiao Cai, Abu Naser Md Ahsanul Haque, Renuka Dhandapani, Maryam Naebe

**Affiliations:** 1 Institute for Frontier Materials, Deakin University, Geelong, VIC 3216, Australia; 2 Cotton Incorporated, 6399 Weston Parkway, Cary, NC 27513, USA

**Keywords:** cotton gin trash, industrial extrusion, circular economy, biodegradable plastic, lignocellulose

## Abstract

Cotton gin trash (CGT), a lignocellulosic waste generated during cotton fibre processing, has recently received significant attention for production of composite bio-plastics. However, earlier studies were limited to either with biodegradable polymers, through small-scale solution-casting method, or using industrially adaptable extrusion route, but with non-biodegradable polymers. In this study, a scale-up production of completely biodegradable CGT composite plastic film with adjustable biodegradation rate is proposed. First using a twin screw extruder, the prepared CGT powder was combined with polycaprolactone (PCL) to form pellets, and then using the compressing moulding, the pellets were transformed into bio-plastic composite films. Hydrophilic polyethylene glycol (PEG) was used as a plasticiser in the mixture and its impact on the biodegradation rate was analysed. The morphology of CGT bio-plastic composite films showed even distribution of CGT powder within the PCL matrix. The CGT incorporation improved the UV resistance, thermal stability, and Young’s modulus of PCL material. Further, the flexibility and mixing properties of the composites were improved by PEG. Overall, this study demonstrated a sustainable production method of CGT bio-plastic films using the whole CGT and without any waste residue produced, where the degradation of the produced composite films can be adjusted to minimise the environmental impact.

## 1. Introduction

The wide use of non-biodegradable plastics in packaging [1], construction [2], textiles [3], and other industries [3,4], and the lack of plastic waste management have caused great disposal problems, environmental pollution and has resulted in climate change [5]. In 2015, 146 million tonnes of plastic packaging was produced with 96.6% non-recycled [3]. Approximately 2 million tonnes of plastic fragments percolate in rivers each year [6], and microplastic particles have been found in water, salt and wild mussel meant for human consumption [7,8], which are harmful due to the large surface area, stability of the particles and containing chemical additives that are toxic [9]. In 2019, the CO_2_ emissions from plastic production into the air was more than 850 million tonnes which is approximately 2% of the global CO_2_ output [10].

One of the long-term goals of the sustainable circular economy is to minimise the environmental impact by making plastic from renewable and biodegradable resources [3]. Examples of the biodegradable resources like natural polymers, such as cellulose [11], lignocellulose [12], and protein [13,14,15] which are abundant and biodegradable.

Cotton gin trash (CGT) is a large quantity of lignocellulosic waste that gets produced in the ginning process during cotton fibre cleaning. This trash contains combination of cotton fibres, cotton burrs, leaves, sticks, broken seeds, and fine particles [16]. In any given year about 8.6 to 21.3 million metric tonnes of CGT are produced worldwide [17]. However, this large pile of waste is usually disposed by sending it to landfills or composting at extra cost.

Natural polymers like CGT are not thermoplastic by nature, hence chemical and physical modifications are often introduced to make them thermoplastic [18]. Chemical modification of lignocellulose has shown some promising results [19]. However, the fabrication method of final material (e.g., plastic film) is usually achieved through solution casting method, therefore, it is industrially less feasible compared to the current extrusion-based processes for preparing commercial plastics. Recently there has been lots of interest in producing bio-based composites by combining natural polymers with different thermoplastic polymers. In this regard, polycaprolactone (PCL), a biodegradable polyester, has received great attention due to its proven biocompatibility and low-temperature processing, which are essential for scale up thermoplastic production [20]. PCL has been blended with natural fibres such as coconut fibres [21], wood flour [22], macaiba fiber [23] and wool [24] to produce sustainable composites. Preparation of PCL composites have been more frequently used in various applications, such as films [25], 3D printing [24], tissue engineering and medical devices [20]. The melting point of PCL is around 60 °C, thus can be conveniently processed and scaled-up by conventional plastic production lines such as hot-pressing [26], blow film extrusion [27], and cast film extrusion [24].

For fabrication of plastics, CGT has been previously combined with different synthetic polymers such as poly(vinyl alcohol) (PVA) [12,28], polypropylene [29], and low-density polyethylene (LDPE) [30]. However, these studies either have used biodegradable polymers through solution casting in small-scale, or industrially more viable method, i.e., extrusion, though with non-biodegradable polymers. Therefore, an applicable way of preparing sustainable and completely biodegradable plastic from CGT is highly needed and there is little if any study available on CGT/PCL combination.

A CGT/PCL combination bio-plastic is likely to biodegrade but the rate of biodegradation is also an important factor for bio-plastics, as often a higher or lower rate can be more suitable based on the particular application of the plastic. For example, the plastics used for single-use are required to quickly degrade when disposed to reduce the load on the environment. However, for some outdoor applications like mulching, plastic needs to be stable during the plant growing season, and later needs to be degraded before the next season starts. Therefore, production of bio-plastics with a control on its degradation rate is useful for preparation of the right material for the right application. In this regard, polyethylene glycol (PEG) could be an interesting choice as a third component for altering the biodegradation rate. PEG is hydrophilic and well-known for its degradation by aerobic and anaerobic bacteria that are naturally abundant in the environment [31]. PEG is also largely produced commercially and widely used in diverse industries. Though PEG has been previously used as a plasticiser for composites, its effectiveness on the biodegradation rate was not evaluated.

Overall, the novelty of this study lies in the combination of using scale-up methods for composite film production, investigating the impact of PEG as a third component, minimising in-process waste, and conducting a comprehensive analysis of various prop-erties of the produced composite films. The morphology, chemical structure, optical transmittance, thermal property, mechanical properties and biodegradation of the produced CGT bio-plastic composite films were investigated.

## 2. Materials and Methods

### 2.1. Materials

CGT powder was obtained by the process as detailed in our previous study [32]. CGT was cut using a rotary cutter mill (Pulverisette 19, Fritsch GmbH, Idar-Oberstein, Germany), followed by 4 h attritor milling (Attritor mill, S/1, Union Process, Akron, OH, USA) and spray drying (spray dryer, B-290, Buchi Labortechnik AG, Flawil, Switzerland). The particle size of the CGT powder thus obtained was around 4.9 µm (D × (50)). Polycaprolactone (PCL, CAPA 6800, Mw 80,000, Melt flow index: 4.03–2.01 g/10 min, Density at 60 °C: 1.1 g/cm³, Era Polymers, Banksmeadow, Australia), and polyethylene glycol (PEG, Mw 10,000, Fluka Chemika, Charlotte, NC, USA) were used as received. Zip lock plastic bag (LDPE) was sourced from local market for comparison purpose.

### 2.2. Fabricating CGT/PCL Bio-Plastic Composite Pellets

**Figure 1:**
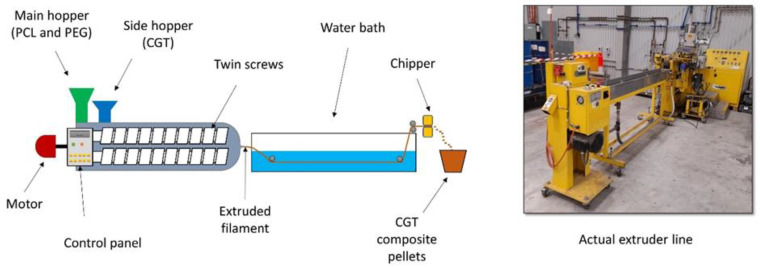
Wayne twin screw extruder (blending) line.

CGT/PCL mixture was produced using a Wayne twin screw extruder (co-rotating, blending) line (Figure 1) (Wayne Machine & Die Co, Richmond, USA). PCL pellets were fed in the main feeding hopper, and the CGT powder was fed through the side feeding hopper. To get a good melting flow of PCL, several trials were carried out and the final optimum temperature was set in the control panel as follows: Zone 1 (88 °C), Zone 2 (90 °C), Zone 3 (90 °C), Zone 4 (93 °C), Zone 5 (96 °C), Zone 6 (98 °C), Die Zone 1 (101 °C), melt temperature (105 °C). The screw speed was set to 150 rpm. After multiple trials, it was found that CGT as high as 40% could be used without a plasticiser to extrude continuous filament from the extruder. When the CGT percentage increased to 50%, the CGT50/PCL50 filament broke into shorter filaments. Thus CGT40/PCL60 mixture was obtained with the main feeding hopper set at a feeding rate of 1.5 kg/h and the side feeding hopper set at a feeding rate of 1 kg/h. CGT/PCL composite filament was formed and then cooled in the water bath. CGT/PCL pellets were collected after chopping the continuous filament into small pellets using the chipper at end of the extruding line.

### 2.3. Fabricating CGT/(PEG/PCL) Bio-Plastic Composite Pellets

PEG flakes were first ground into powder using a Ring Grinder (Variable Speed Rotor Mill, Pulverisette 14, Fritsch, Germany), then melt blended with PCL and extruded into PEG/PCL mixture pellets with two different ratios PEG10/PCL90 and PEG20/PCL80. 50% CGT was the highest percentage that the CGT/(PEG/PCL) composite can be produced with continuous filament. CGT50/(PEG10PCL90)50 and CGT50/(PEG20PCL80)50 pellets were produced as follows: PEG/PCL mixture pellets were fed in the main feeding hopper, and CGT powder was fed in the side feeding hopper. The feeding rates of these two hoppers were adjusted based on the weight ratio of CGT as described in Section 2.2. The temperature was set in the control panel as mentioned in Section 2.2.

### 2.4. Fabricating CGT Bio-Plastic Composite Films Using Compression Moulding

CGT/(PEG/PCL) composite pellets were spread evenly in the metallic mould, which was put in the hot press machine (McMillan Engineering Group, Dandenong South, Australia) and heated at 80 °C for 20 min to melt the pellets. Then the press was applied using 6.3 t force, and the temperature gradually increased step by step. The press was applied to the melted pellets (80 °C) initially for 20 min, followed by at 90 °C for 10 min, at 100 °C for another 10 min and finally at 105 °C for 10 min. 100% PCL film as a control sample was also fabricated using the same conditions. Film thickness was measured using a digital outside micrometer (ACCUD, Suzhou, China).

### 2.5. Characterisations

#### 2.5.1. Morphology

The surface and cross-section morphology of CGT bio-plastic composite films were observed using scanning electron microscopy (SEM) (Zeiss Supra 55VP, Carl Zeiss, Jena, Germany). Films were first gold sputter-coated by Leica EM ACE600 and then imaged using the SEM with 15 kV accelerating voltage.

#### 2.5.2. Chemical Structure

The Fourier-Transform Infrared (FTIR) spectra of the CGT powder, PCL pellet, PCL film after degradation, PEG flakes, and CGT bio-plastic composite films (CGT40/PCL60 (before and after degradation), CGT50/(PEG10/PCL90)50 (before and after degradation), and CGT50/(PEG20/PCL80)50 were measured by Bruker Vertex 70 FTIR spectrometer with an ATR (attenuated total reflectance) mode, a scan resolution of 4 cm^−1^ and 64 scans per sample in the range of 4000 cm^−1^–500 cm^−1^. The spectral data were analysed (baseline correction) using OPUS 5.5 software (Bruker Corporation, Billerica, MA, USA).

#### 2.5.3. Optical Transmittance

The transmittance of the bio-plastic films, PCL film and a commercial low-density polyethylene film (LDPE, sourced from a ziplock plastic bag) were measured in the transmittance mode using a UV-Vis-NIR spectrophotometer (Cary 5000 Scan, Varian Inc., Palo Alto, CA, USA) in the range of 200 nm to 800 nm.

#### 2.5.4. Thermal Property

Thermogravimetric analysis (TGA) of CGT powder, PCL pellet, PEG flakes and CGT bio-plastic composite films were tested using TGA Q50 thermal analyser (TA instruments, New Castle, DE, USA). Samples (around 10 mg) were heated from 30 °C to 500 °C with a heating rate of 10 °C min^−1^ under the nitrogen atmosphere (Balance Gas of Nitrogen at 40.0 mL/min, and Sample Gas of Nitrogen at 60.0 mL/min).

#### 2.5.5. Mechanical Properties

The Young’s modulus, tensile strength and elongation at break of the bio-plastic films, PCL film, and the commercial LDPE were measured using a universal tensile testing system (Instron 5967, USA) with a 100 N load cell at a constant elongation rate of 50 mm/min. Standard test method for tensile properties of thin plastic sheeting (D882–18) was used as a reference and the test parameters were modified based on the fabricated samples. The yield point is the first point on the stress-strain curve at which an increase in strain occurs without an increase in stress. Tensile strength and elongation at the yield point of the developed bio-plastic films, PCL film, and the commercial LDPE were also analysed.

#### 2.5.6. Biodegradation

The CGT bio-plastic composite films and 100% PCL film were cut into squared shapes (1 cm × 1 cm) and buried in potting mix soil which complies with the requirement of AS4454 for a premium-grade potting mix in a container. All film samples were prepared in triplicate and buried in the soil at each time point using different containers. The containers were kept in an incubator at 30 °C for 2 months. Water was sprayed on the soil in the containers once every 3 days to make up for the gradual water evaporation. Samples were taken out, cleaned and weighed every month to calculate the weight loss (%) by the degradation. Dried samples were also scanned using SEM to investigate the film morphology after biodegradation. Physical changes of PCL and CGT bio-plastic composite films were recorded after 1 month and 2 months of biodegradation in soil.

## 3. Results

### 3.1. Fabrication of CGT Bio-Plastic Composite Pellets and Bio-Plastic Films

**Figure 2:**
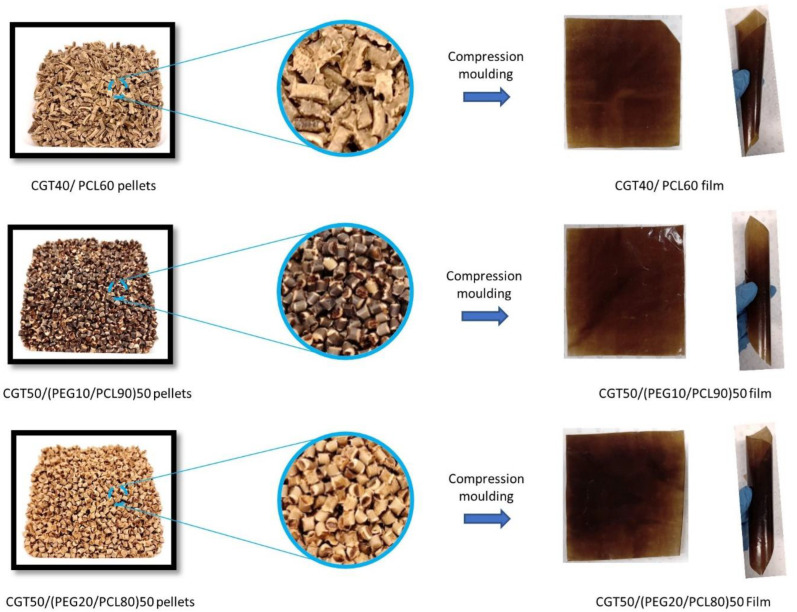
CGT bio-plastic composite pellets and films.

CGT bio-plastic composite pellets and films are shown in Figure 2. CGT at 40% is the highest concentration that can be successfully used to make smooth and continuous CGT/PCL filament that can be chopped into uniform sized composite pellets. To increase the CGT content beyond 40% in the composite, a plasticiser (i.e., PEG) was needed to improve the interactions between CGT and PCL. Plasticisers have been frequently added into polymer composites due to their improvement in composite processibility, ductility and flexibility [33]. Earlier research has found that PEG helped improve the interfacial interaction between polylactide (PLA) and thermoplastic starch [34]. It has also been reported that PEG improved not only the film flexibility but also enhanced its mechanical properties [33,35]. Further, due to the strong hydrophilic groups in PEG, microorganisms easily colonised on the films which further helped in biodegradation of the films. In this study, 10% and 20% PEG were melt-blended with PCL for the fabrication of CGT50/(PEG10/PCL90)50, and CGT50/(PEG20/PCL80)50. Addition of PEG as plasticiser facilitated to increase the percentage of CGT from 40% to 50% in the composite films. There were colour differences among three different CGT bio-plastic composite pellets, which could be attributed to the variation of CGT and the addition of different percentages of PEG. However, the three produced CGT bio-plastic composite films showed similar dark brown colours. CGT40/PCL60 film showed the similar flexibility and colour consistency as the CGT bio-plastic composite films with different percentages of plasticiser. The thickness of the bio-plastic films was around 105 ± 10 µm.

### 3.2. CGT Bio-Plastic Composite Film Morphology

**Figure 3:**
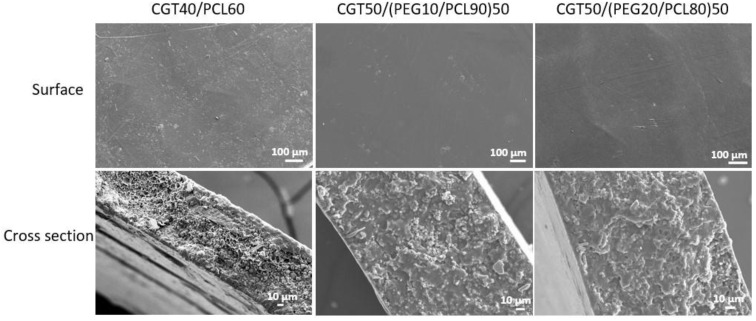
Scanning electron microscopic (SEM) images of the CGT bio-plastic composite films.

The morphology of CGT bio-plastic composite films’ surface and cross-section are as shown in Figure 3. The surface of the three types of CGT bio-plastic composite films was relatively smooth. The CGT powders (40%) got distributed evenly and mixed well with the PCL (60%) as shown in the CGT40/PCL60 cross-section image. When CGT content increased to 50%, the addition of plasticiser PEG improved the miscibility of CGT and PCL as shown in the cross-section images indicating the PEG acting as a compatibiliser between CGT and PCL.

### 3.3. Chemical Structure

**Figure 4:**
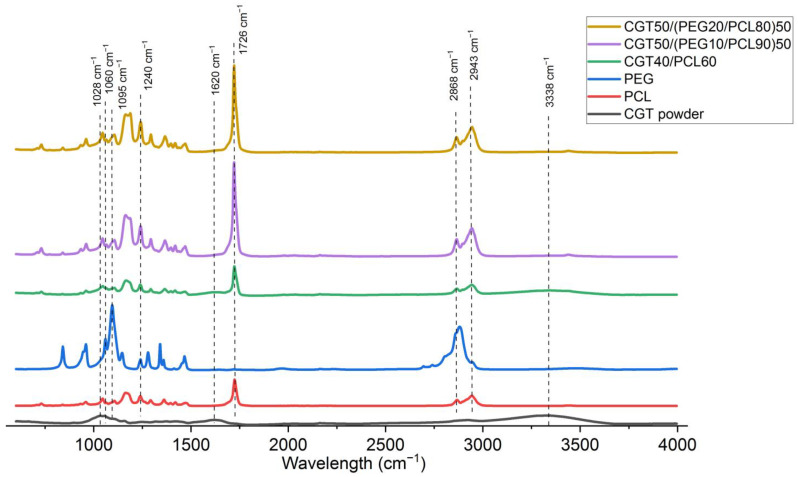
Fourier-transform infrared spectroscopy (FTIR) spectra of CGT powder, PCL pellet, PEG flakes, and CGT bio-plastic composite films (CGT40/PCL60, CGT50/(PEG10/PCL90)50, and CGT50/(PEG20/PCL80)50).

Using the FTIR spectroscopy, the chemical structure of CGT bio-plastic composite films both with and without PEG were measured as shown in Figure 4. A broad peak around 3338 cm^−1^ in the CGT powder indicated the O–H stretching vibration corresponding to the lignocellulose structure [36]. This peak was shifted to 3446 cm^−1^, 3438 cm^−1^, and 3440 cm^−1^ for CGT40/PCL60, CGT50/(PEG10/PCL90)50, and CGT50/(PEG20/PCL80)50, respectively, which could be due to the formation of intermolecular hydrogen bonding between CGT and polymers (PCL and PEG) [37]. The peaks between 1028–1060 cm^−1^ in both the CGT powder and CGT bio-plastic composite films were assigned to the corresponding peaks of cellulose (C–O–C stretching vibrations) [12]. The corresponding peak for lignin (C=C stretching) at around 1620 cm^−1^ was observed in both the CGT powder and CGT bio-plastic composite films. For PCL both symmetric and asymmetric CH2 stretching at 2868 cm^−1^ and 2943 cm^−1^ and carbonyl stretching (around 1726 cm^−1^) [38] were observed in all three types of CGT bio-plastic composite films. The C–O stretching of alcohol around 1240 cm^−1^ [39] was found in PEG, PCL, and all three types of CGT bio-plastic composite films. The peak around 1095 cm^−1^ was assigned to C–O–C ether in PEG [39]. All the relevant peaks of each single component were observed in the CGT bio-plastic composite films, which indicated a good mixing of these components. There were no new peaks in the spectra of CGT bio-plastic composite films, which indicated the CGT bio-plastic composite films did not form any functional groups during processing and only interacted together at the molecular level [37].

### 3.4. Optical Transmittance

**Figure 5:**
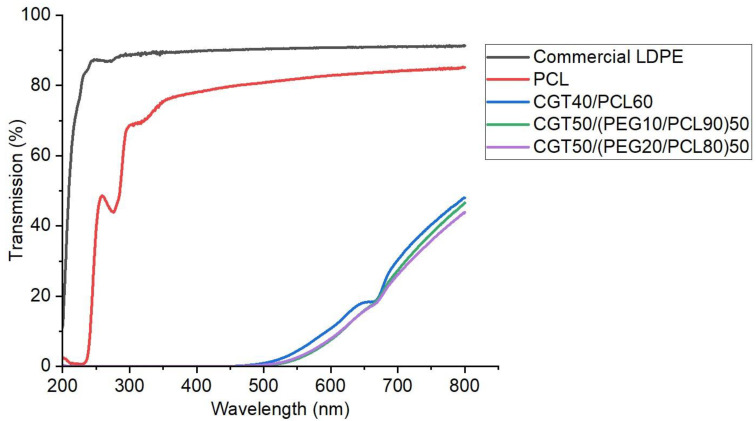
Transmission spectra of the commercial LDPE, PCL and CGT bio-plastic composite films.

The optical properties of the commercial LDPE, PCL film and CGT bio-plastic composite films under UV (200–400 nm) and visible (400–800 nm) light are shown in Figure 5. The commercial LDPE and PCL film showed high visible light transparency (around 90% and 80%, respectively) due to its low absorbance in the visible light range (400 nm to 800 nm). The transmission for commercial LDPE and PCL films at 200 nm was 11.2% and 2.3% respectively. Around 275 nm due to the absorption of UV light a band was also observed in both the films. All three types of CGT bio-plastic composite films showed decreased visible light transmission with the light wavelength reduced from 800 nm to 450 nm, and at high ratio of CGT in the bio-plastic composite film showed a low light transmission due to the brown colour of CGT [12]. This was shown as a band near 680 nm, which was attributed to the existence of lignin in CGT that contained many chromophore groups such as guaiacyl (yellow-brown), syringyl (red-purple) and aromatic rings [40]. The UV light from 400 nm to 200 nm showed 0% UV light transmission in all three types of CGT bio-plastic composite films, which was attributed to the existence of lignin in CGT-containing phenolic groups [28]. This zero UV transmission (UV shielding properties) as shown by the CGT bio-plastic composite films can be useful for some applications such as mulch film to retain moisture and prevent weeds growth [41].

### 3.5. Thermal Property

**Figure 6:**
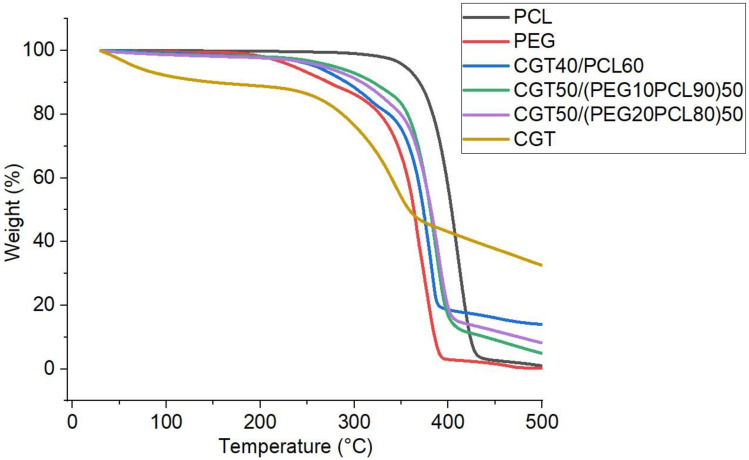
Thermogravimetric behaviour of CGT powder, PCL pellet, PEG powder, CGT40/PCL60 film, CGT50/(PEG10PCL90)50 film, and CGT50/(PEG20PCL80)50 film.

The thermogravimetric analysis peaks for PCL pellet, PEG flakes, CGT powder, CGT40/PCL60 film, CGT50/(PEG10PCL90)50 film, and CGT50/(PEG20PCL80)50 film are as given in Figure 6. Temperature at 10% and 50% weight loss for each material is listed in Table 1. For CGT powder the weight loss occurred around 30–100 °C due to its moisture content, and the actual degradation for the powder started around 240 °C which can be attributed to its content including cellulose and hemicellulose [42]. PEG started degrading around 165 °C and lost 97% weight at 390 °C, while PCL started degrading around 270 °C and lost 97% weight at 440 °C. CGT bio-plastic composite films started degrading from around 220 °C due to their contents including CGT, PCL and PEG. 50% weight loss of CGT40/PCL60, CGT50/(PEG10PCL90)50, CGT50/(PEG20PCL80)50 and CGT powder occurred at 374 °C, 381 °C, 382 °C, and 359 °C, respectively, which was probably due to the formation of intermolecular reactions between CGT and polymer (PCL and PEG) [43]. Both PEG and PCL degraded completely at 500 °C. The bio-plastic films degraded about 81% around 390 °C and there was still some weight left at 500 °C due to the complex contents in CGT such as lignin, inorganic materials, and ashes [28].

### 3.6. Mechanical Property

**Figure 7:**
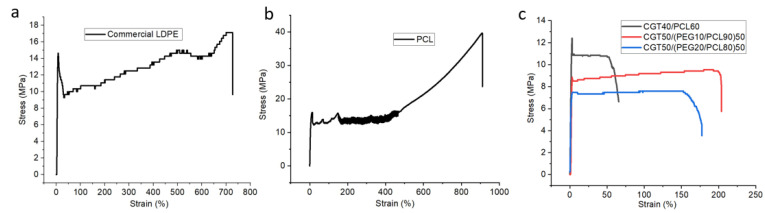
Representative stress-strain curves of (**a**) the commercial LDPE, (**b**) PCL, and (**c**) CGT bio-plastic composite films.

The representative stress-strain curves of the commercial LDPE, PCL and CGT bio-plastic composite films are as illustrated in Figure 7, which also shows their yield point and break point. The Young’s modulus, tensile strength, and elongation at yield point and breaking point for all the films are as listed in Table 2. The Young’s modulus of CGT40/PCL60 was 563.7 ± 56.1 MPa, which is around 1.9 times higher than the commercial LDPE (289.9 ± 48.7 MPa) and about twice higher than the PCL film (275.4 ± 6.0 MPa). While the Young’s modulus of CGT50/(PEG10/PCL90)50 and CGT50/(PEG20/PCL80)50 bio-plastic films was around 1.6 times higher than that of commercial LDPE and PCL films, respectively. However, the elongation and tensile strength for the new films at the yield point and break point were lower than the commercial LDPE and PCL films. This can be attributed to the higher modulus and lower elongation property of cellulose [12,28] present in CGT. The elongation at the breaking point for CGT40/PCL60 film was 42.5 ± 27.7%. However, the elongation at break for CGT50/(PEG10/PCL90)50 and CGT50/(PEG20/PCL80)50 films was 198.0 ± 66.5% and 140.6 ± 52.2%, respectively. This increase in elongation can be attributed to the addition of plasticiser PEG (i.e., increase in elongation and reduction in strength and modulus) [12], thus producing flexible films. Therefore, the flexibility of the CGT bio-plastic composite films increased with the addition of PEG, i.e., the elongation at break of CGT50/(PEG10/PCL90)50 and CGT50/(PEG20/PCL80)50 increased 4.7 and 3.3 times respectively when compared to that of CGT40/PCL60; and their modulus were lower than that without a plasticiser. However, when the amount of PEG increased in the bio-plastic film, the elongation and tensile strength at the breaking point decreased as shown between CGT50/(PEG20/PCL80)50 and CGT50/(PEG10/PCL90)50. A similar trend was also reported by another researcher where PEG content greater than 15% in polylactide/poly(ethylene glycol) blends showed a decreased tensile strain at break [33]. Therefore, the mechanical properties of the bio-plastic film can be optimised for different applications by varying the amount of PEG in the recipe.

### 3.7. Biodegradation of CGT/PCL Bio-Plastic Composite Films

**Figure 8:**
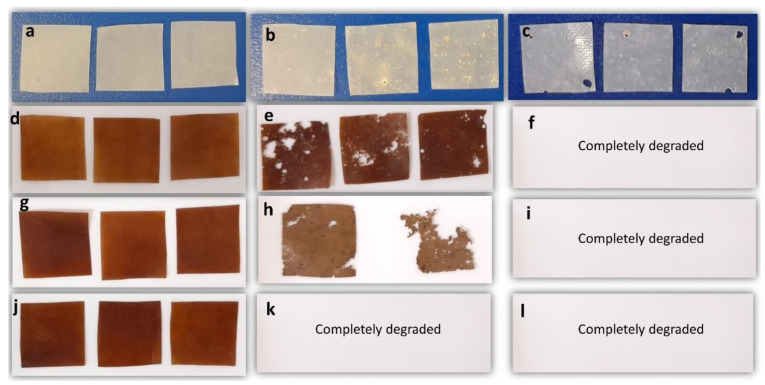
The physical changes of PCL film and CGT bio-plastic composite films before and after soil degradation. PCL film before (**a**), after 1 month (**b**) and 2 months (**c**) soil biodegradation; CGT40/PCL60 film before (**d**), after 1 month (**e**) and 2 months (**f**) soil biodegradation; CGT50/(PEG10/PCL90)50 film before (**g**), after 1 month (**h**) and 2 months (**i**) soil biodegradation; CGT50/(PEG20/PCL80)50 film before (**j**), after 1 month (**k**) and 2 months (**l**) soil biodegradation.

**Figure 9:**
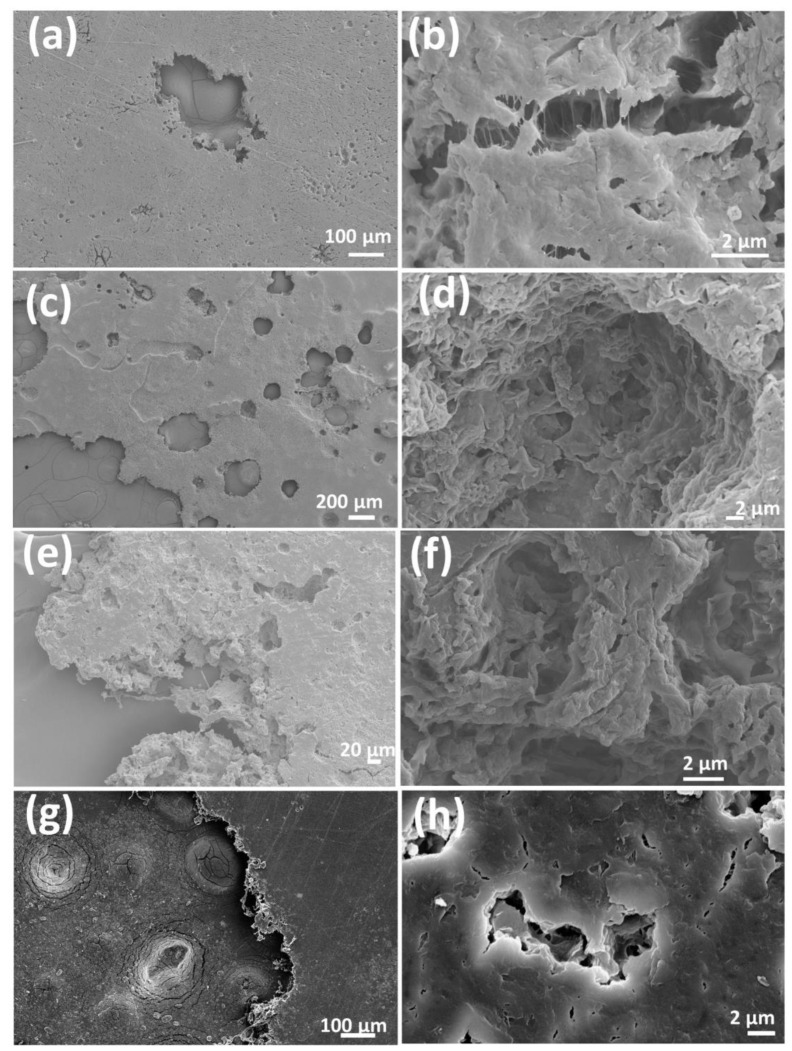
SEM images of PCL and CGT bio-plastic composite film surface after months of biodegradation in soil: (**a**,**b**) PCL film after 1-month biodegradation, (**c**,**d**) CGT40/PCL60 film after 1 month biodegradation, (**e**,**f**) CGT50/(PEG10/PCL90)50 film after 1 month biodegradation, and (**g**,**h**) PCL film after 2 months of biodegradation.

**Figure 10:**
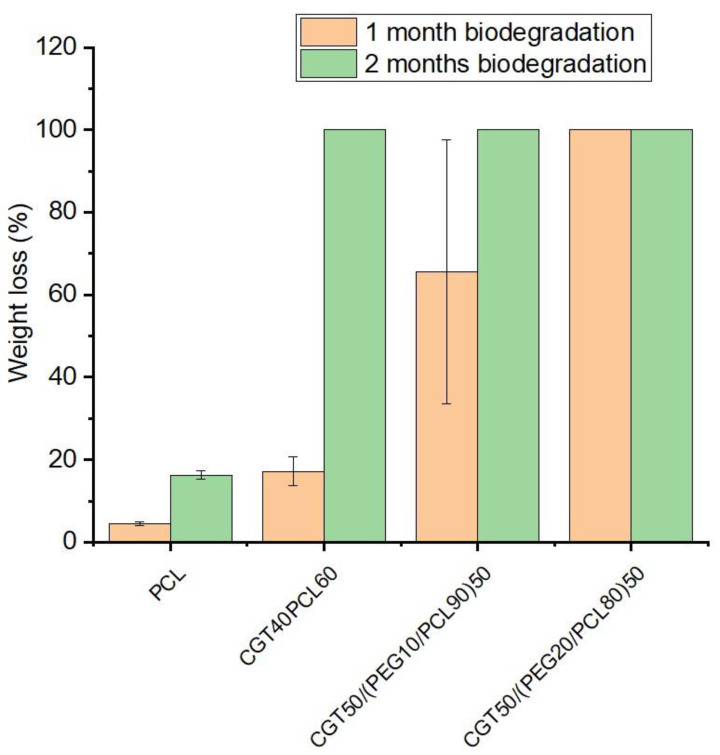
Weight loss of PCL and CGT bio-plastic composite films after first and second month of biodegradation in soil.

**Figure 11:**
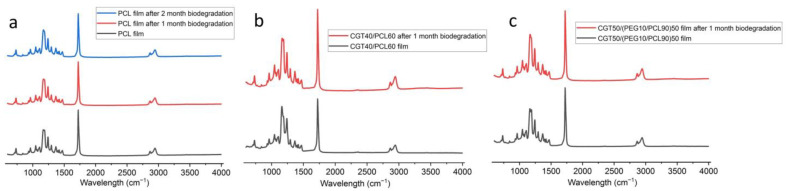
Fourier-transform infrared spectroscopy (FTIR) spectra of (**a**) PCL film, (**b**) CGT40/PCL60 and (**c**) CGT50/(PEG10/PCL90)50 composite film before and after biodegradation in soil.

Physical changes of PCL and CGT bio-plastic composite films over a period of 2 months of biodegradation in soil are as shown in Figure 8. After 1 month in soil, due to biodegradation, rough and eroded surface with small pores was seen on PCL films. CGT40/PCL60 and CGT50/(PEG10/PCL90)50 bio-plastic films lost their initial appearance and structural integrity and had rough and porous surfaces. Among the three samples from CGT50/(PEG10/PCL90)50 bio-plastic film, one sample had completely degraded at this point (Figure 8h). The most interesting fact was that all the three samples of CGT50/(PEG20/PCL80)50 bio-plastic composite film completely degraded within the first month of being buried in the soil (Figure 8k). The results obtained in this study indicate a considerable level of soil biodegradation of the new CGT composite films by the microorganisms present in the soil [44]. After 2 months of being buried in soil, all the CGT bio-plastic composite films were completely degraded, while the PCL films showed rough, eroded surfaces, and large pores on its surface (Figure 8c). Though both CGT40/PCL60 and CGT50/(PEG10/PCL90)50 samples degraded within two months, the rate of degradation for the PEG containing sample was probably faster as perceived from the photographs of the samples taken after first month of being buried in the soil. This result indicates that the amount of PEG added to the formulation can be sensibly used to control the biodegradation rate of CGT/PCL bio-plastic composite films. The morphological and structural changes that occurred in the composite films were confirmed by SEM with pores and cracks observed in the films as shown in Figure 9. The weight loss calculated for the PCL and CGT bio-plastic composite films due to biodegradation is shown in Figure 10. PCL films lost around 4% weight after first month of biodegradation and the weight loss reached around 16% after second month of biodegradation. CGT40/PCL60 film had around 17% and 100% weight loss respectively after first and second month of biodegradation. With the addition of the plasticiser (PEG), the CGT bio-plastic composite films showed higher amounts of weight loss after first month (around 65% and 100% for CGT50/(PEG10/PCL90)50 and CGT50/(PEG20/PCL80)50, respectively). This could be attributed to the hydrophilic property of PEG helping film degradation under warm and humid conditions [45,46], which has been reported that the hydrophilicity of PEG increased the degradation of Poly(butylene adipate-co-terephthalate) [46], and the biodegradation kinetics of PEG was accelerated with the increasing of temperature from 20 °C to 30 °C. There were no chemical structure changes after degradation in soil as analysed by FTIR, illustrated in Figure 11.

## 4. Conclusions

In this study, bio-plastic films from different combinations including CGT, PCL and PEG (CGT40/PCL60, CGT50/(PEG10/PCL90)50 and CGT50/(PEG20/PCL80)50) were successfully produced using a scale-up production line without any waste residue. Properties of CGT bio-plastic composite films and 100% PCL were investigated including films’ morphology, mechanical, optical, thermal and biodegradation properties. Further the mechanical and optical properties were also compared with a commercial LDPE plastic (a ziplock plastic bag). CGT bio-plastic composite films showed higher Young’s modulus than both the LDPE and PCL films, while their elongation and tensile strength at the yield point and break point were lower than the LDPE and PCL films. CGT bio-plastic composite films with PEG showed improved flexibility and increased elongation at break (more than 3 times higher) and decreased modulus compared with sample without PEG. All CGT bio-plastic composite films showed improved thermal stability compared with PCL and PEG, higher biodegradation rates when compared to PCL and better UV resistance compared to pure PCL and LDPE. When the amount of PEG increased from 10% to 20%, the mechanical properties, such as elongation and tensile strength decreased, while the biodegradation rate improved. Therefore, CGT bio-plastic composite films with a higher amount of PEG can be ideal where strength requirement is only secondary to a faster degradation rate (such as single use packaging). While CGT bio-plastic composite films with a lower amount of PEG can be useful where more stability is required during the use (such as mulching film). Thus, the properties of CGT bio-plastic composite films can be customised and adjusted depending on specific requirement in relation to their strength, flexibility and biodegradation properties. Further, the proof of large-scale production of biodegradable CGT bio-plastic composite films in this study could be encouraging for its industrial production in near future.

## Figures and Tables

**Table 1:** Temperature at 10% and 50% weight loss for each material.

	Temperature at 10% Weight Loss (°C)	Temperature at 50% Weight Loss (°C)
PCL	369	403
PEG	273	363
CGT40PCL60	292	374
CGT50/(PEG10/PCL90)50	320	381
CGT50/(PEG20/PCL80)50	309	382
CGT	152	359

**Table 2:** Mechanical properties of commercial LDPE, PCL, and CGT bio-plastic composite films.

Samples	Young’s Modulus (MPa)	Tensile Strength at Yield (MPa)	Elongation at Yield (%)	Tensile Strength at Break (MPa)	Elongation at Break (%)
Commercial LDPE	289.9 ± 48.7	13.7 ± 0.7	10.3 ± 2.0	17.5 ± 0.3	746.8 ± 82.6
PCL	275.4 ± 6.0	14.7 ± 1.5	12.9 ± 1.4	40.2 ± 6.0	900.8 ± 88.7
CGT40/PCL60	563.7 ± 56.1	10.9 ± 1.6	3.2 ± 0.2	9.8 ± 1.4	42.5 ± 27.7
CGT50/(PEG10/PCL90)50	464.3 ± 61.1	7.6 ± 1.1	2.6 ± 0.4	9.2 ± 0.5	198.0 ± 66.5
CGT50/(PEG20/PCL80)50	478.5 ± 65.2	6.8 ± 1.1	3.0 ± 1.0	7.4 ± 0.1	140.6 ± 52.2

## Data Availability

The data presented in this study are available on request from the corresponding author.

## References

[1] Geyer R., Jambeck J.R., Law K.L. (2017). Production, use, and fate of all plastics ever made. Sci. Adv..

[2] Kunthawatwong R., Sylisomchanh L., Pangdaeng S., Wongsa A., Sata V., Sukontasukkul P., Chindaprasirt P. (2022). Recycled Non-Biodegradable polyethylene terephthalate waste as fine aggregate in fly ash geopolymer and cement mortars. Constr. Build. Mater..

[3] Rosenboom J.-G., Langer R., Traverso G. (2022). Bioplastics for a circular economy. Nat. Rev. Mater..

[4] Cai Z., Fan L., Wang H., Lamon S., Alexander S.E., Lin T., Edwards S.L. (2021). Constructing 3D Macroporous Microfibrous Scaffolds with a Featured Surface by Heat Welding and Embossing. Biomacromolecules.

[5] Garrido T., Etxabide A., Guerrero P., De la Caba K. (2016). Characterization of agar/soy protein biocomposite films: Effect of agar on the extruded pellets and compression moulded films. Carbohydr. Polym..

[6] Jambeck J.R., Geyer R., Wilcox C., Siegler T.R., Perryman M., Andrady A., Narayan R., Law K.L. (2015). Plastic waste inputs from land into the ocean. Science.

[7] Kosuth M., Mason S.A., Wattenberg E.V. (2018). Anthropogenic contamination of tap water, beer, and sea salt. PLoS ONE.

[8] Catarino A.I., Macchia V., Sanderson W.G., Thompson R.C., Henry T.B. (2018). Low levels of microplastics (MP) in wild mussels indicate that MP ingestion by humans is minimal compared to exposure via household fibres fallout during a meal. Environ. Pollut..

[9] Ziccardi L.M., Edgington A., Hentz K., Kulacki K.J., Kane Driscoll S. (2016). Microplastics as vectors for bioaccumulation of hydrophobic organic chemicals in the marine environment: A state-of-the-science review. Environ. Toxicol. Chem..

[10] Hamilton L.A., Feit S. (2019). Plastic & Climate: The Hidden Costs of a Plastic Planet.

[11] Tu H., Zhu M., Duan B., Zhang L. (2021). Recent progress in high-strength and robust regenerated cellulose materials. Adv. Mater..

[12] Haque A.N.M.A., Naebe M. (2021). Flexible water-resistant semi-transparent cotton gin trash/poly (vinyl alcohol) bio-plastic for packaging application: Effect of plasticisers on physicochemical properties. J. Clean. Prod..

[13] Cai Z., Al Faruque M., Kiziltas A., Mielewski D., Naebe M. (2021). Sustainable lightweight insulation materials from textile-based waste for the automobile industry. Materials.

[14] Fan L., Li J.-L., Cai Z., Wang X. (2018). Creating biomimetic anisotropic architectures with co-aligned nanofibers and macrochannels by manipulating ice crystallization. ACS Nano.

[15] Fan L., Li J.-L., Cai Z., Wang X. (2021). Bioactive hierarchical silk fibers created by bioinspired self-assembly. Nat. Commun..

[16] Haque A.N.M.A., Remadevi R., Naebe M. (2021). A review on cotton gin trash: Sustainable commodity for material fabrication. J. Clean. Prod..

[17] Haque A.N.M.A., Remadevi R., Wang X., Naebe M. (2020). Adsorption of anionic Acid Blue 25 on chitosan-modified cotton gin trash film. Cellulose.

[18] Müller K., Zollfrank C., Schmid M. (2019). Natural polymers from biomass resources as feedstocks for thermoplastic materials. Macromol. Mater. Eng..

[19] Xia Q., Chen C., Yao Y., Li J., He S., Zhou Y., Li T., Pan X., Yao Y., Hu L. (2021). A strong, biodegradable and recyclable lignocellulosic bioplastic. Nat. Sustain..

[20] Tran T.N., Bayer I.S., Heredia-Guerrero J.A., Frugone M., Lagomarsino M., Maggio F., Athanassiou A. (2017). Cocoa shell waste biofilaments for 3D printing applications. Macromol. Mater. Eng..

[21] Priselac D., Mahović Poljaček S., Tomašegović T., Leskovac M. (2022). Blends based on poly (ε-Caprolactone) with addition of poly (lactic acid) and coconut fibers: Thermal analysis, ageing behavior and application for embossing process. Polymers.

[22] Barreto Luna C.B., dos Santos Filho E.A., Siqueira D.D., de Souza D.D., Ramos Wellen R.M., Araújo E.M. (2022). Jatobá wood flour: An alternative for the production of ecological and sustainable PCL biocomposites. J. Compos. Mater..

[23] Siqueira D., Luna C., Ferreira E., Araújo E., Wellen R. (2020). Tailored PCL/Macaíba fiber to reach sustainable biocomposites. J. Mater. Res. Technol..

[24] Haque A.N.M.A., Naebe M., Mielewski D., Kiziltas A. (2023). Waste wool/polycaprolactone filament towards sustainable use in 3D printing. J. Clean. Prod..

[25] Lyu J.S., Lee J.-S., Han J. (2019). Development of a biodegradable polycaprolactone film incorporated with an antimicrobial agent via an extrusion process. Sci. Rep..

[26] Peng X., Zhang Z. (2018). Hot-pressing composite curling deformation characteristics of plastic film-reinforced pliable decorative sliced veneer. Compos. Sci. Technol..

[27] Thakur M., Majid I., Hussain S., Nanda V. (2021). Poly (ε-caprolactone): A potential polymer for biodegradable food packaging applications. Packag. Technol. Sci..

[28] Haque A.N.M.A., Remadevi R., Wang X., Naebe M. (2020). Biodegradable cotton gin trash/poly (vinyl alcohol) composite plastic: Effect of particle size on physicochemical properties. Powder Technol..

[29] Ge C., Cheng H.N., Miri M.J., Hailstone R.K., Francis J.B., Demyttenaere S.M., Alharbi N.A. (2020). Preparation and evaluation of composites containing polypropylene and cotton gin trash. J. Appl. Polym. Sci..

[30] Cheng H., Dowd M., Finkenstadt V., Selling G., Evangelista R., Biswas A. (2013). Use of cotton gin trash and compatibilizers in polyethylene composites. Green Polymer Chemistry: Biocatalysis and Materials II.

[31] Rosli N.A., Karamanlioglu M., Kargarzadeh H., Ahmad I. (2021). Comprehensive exploration of natural degradation of poly (lactic acid) blends in various degradation media: A review. Int. J. Biol. Macromol..

[32] Cai Z., Haque A.N.M.A., Dhandapani R., Naebe M. (2022). Impact of variability of cotton gin trash on the properties of powders prepared from distinct mechanical approaches. Powder Technol..

[33] Li F.-J., Liang J.-Z., Zhang S.-D., Zhu B. (2015). Tensile properties of polylactide/poly (ethylene glycol) blends. J. Polym. Environ..

[34] Ferrarezi M.M.F., de Oliveira Taipina M., Escobar da Silva L.C., Gonçalves M.d.C. (2013). Poly (ethylene glycol) as a compatibilizer for poly (lactic acid)/thermoplastic starch blends. J. Polym. Environ..

[35] Salahuddin Z., Farrukh S., Hussain A. (2018). Optimization study of polyethylene glycol and solvent system for gas permeation membranes. Int. J. Polym. Anal. Charact..

[36] Haque A.N.M.A., Remadevi R., Rojas O.J., Wang X., Naebe M. (2020). Kinetics and equilibrium adsorption of methylene blue onto cotton gin trash bioadsorbents. Cellulose.

[37] Vinodhini P.A., Sangeetha K., Thandapani G., Sudha P., Jayachandran V., Sukumaran A. (2017). FTIR, XRD and DSC studies of nanochitosan, cellulose acetate and polyethylene glycol blend ultrafiltration membranes. Int. J. Biol. Macromol..

[38] Mohandesnezhad S., Pilehvar-Soltanahmadi Y., Alizadeh E., Goodarzi A., Davaran S., Khatamian M., Zarghami N., Samiei M., Aghazadeh M., Akbarzadeh A. (2020). In vitro evaluation of Zeolite-nHA blended PCL/PLA nanofibers for dental tissue engineering. Mater. Chem. Phys..

[39] Pramono E., Utomo S., Wulandari V., Clegg F. (2016). FTIR studies on the effect of concentration of polyethylene glycol on polimerization of Shellac. Proceedings of the 8th International Conference on Physics and its Applications (ICOPIA).

[40] Zhang Y., Naebe M. (2021). Lignin: A review on structure, properties, and applications as a light-colored UV absorber. ACS Sustain. Chem. Eng..

[41] Sirivechphongkul K., Chiarasumran N., Saisriyoot M., Thanapimmetha A., Srinophakun P., Iamsaard K., Lin Y.-T. (2022). Agri-Biodegradable Mulch Films Derived from Lignin in Empty Fruit Bunches. Catalysts.

[42] Cai Z., Remadevi R., Al Faruque M.A., Setty M., Fan L., Haque A.N.M.A., Naebe M. (2019). Fabrication of a cost-effective lemongrass (Cymbopogon citratus) membrane with antibacterial activity for dye removal. RSC Adv..

[43] Wang Y., Chen H., Cui L., Tu C., Yan C., Guo Y. (2022). Toughen and strengthen alginate fiber by incorporation of polyethylene glycol grafted cellulose nanocrystals. Cellulose.

[44] Das S., Pandey P., Mohanty S., Nayak S.K. (2017). Evaluation of biodegradability of green polyurethane/nanosilica composite synthesized from transesterified castor oil and palm oil based isocyanate. Int. Biodeterior. Biodegrad..

[45] Sajjan A.M., Naik M.L., Kulkarni A.S., Rudgi U.F.-E.-H., Ashwini M., Shirnalli G.G., Sharanappa A., Kalahal P.B. (2020). Preparation and characterization of PVA-Ge/PEG-400 biodegradable plastic blend films for packaging applications. Chem. Data Collect..

[46] Diao X., Zhang C., Weng Y. (2022). Properties and degradability of poly (butylene adipate-co-terephthalate)/calcium carbonate films modified by polyethylene glycol. Polymers.

